# Isolation and Characterization of Efficient Active Compounds Using High-Performance Centrifugal Partition Chromatography (CPC) from Anti-Inflammatory Activity Fraction of *Ecklonia maxima* in South Africa

**DOI:** 10.3390/md20080471

**Published:** 2022-07-23

**Authors:** Hyun-Soo Kim, Jun-Geon Je, Hyesuck An, Kyunghwa Baek, Jeong Min Lee, Mi-Jin Yim, Seok-Chun Ko, Ji-Yul Kim, Gun-Woo Oh, Min-Cheol Kang, Young Min Ham, You-Jin Jeon, Dae-Sung Lee

**Affiliations:** 1 National Marine Biodiversity Institute of Korea, 75, Jangsan-ro 101 gil, Janghang-eup, Seocheon 33662, Korea; gustn783@mabik.re.kr (H.-S.K.); mgran@mabik.re.kr (H.A.); kyunghwabaek@mabik.re.kr (K.B.); lshjm@mabik.re.kr (J.M.L.); mjyim@mabik.re.kr (M.-J.Y.); seokchunk@mabik.re.kr (S.-C.K.); jiyul2224@mabik.re.kr (J.-Y.K.); ogwchobo@mabik.re.kr (G.-W.O.); 2 Department of Marine Life Science, School of Marine Biomedical Sciences, Jeju National University, Jeju 63243, Korea; wpwnsrjs3@jejunu.ac.kr; 3 Research Group of Food Processing Research Division of Strategic Food Technology, Wanju-gun 55365, Korea; mckang@kfri.re.kr; 4 Korea Jeju Biodiversity Research Institute, Jeju Technopark, Jeju 63608, Korea; hijel@jejutp.or.kr

**Keywords:** *Ecklonia maxima*, high-performance centrifugal partition chromatography, eckmaxol, dieckol, anti-inflammatory, zebrafish

## Abstract

*Ecklonia maxima* is a brown seaweed, which is abundantly distributed in South Africa. This study investigated an efficient approach using high-performance centrifugal partition chromatography (HPCPC), which has been successfully developed for the isolation and purification of phlorotannins, eckmaxol, and dieckol from the ethyl acetate fraction of *E. maxima* (EEM). We evaluated EEM for its inhibitory effect against lipopolysaccharide (LPS)-induced inflammatory responses in zebrafish embryos. The separation of eckmaxol and dieckol from samples of EEM using HPCPC was found to be of high purity and yield under an optimal solvent system composed of n-hexane:ethyl acetate:methanol:water (2:7:3:7, *v*/*v*/*v*/*v*). To evaluate the anti-inflammatory efficacy of EEM containing active compounds, zebrafish embryos exposed to LPS were compared with and without EEM treatment for nitric oxide (NO) production, reactive oxygen species (ROS) generation, and cell death two days after fertilization. These evaluations indicate that EEM alleviated inflammation by inhibiting cell death, ROS, and NO generation induced by LPS treatment. According to these results, eckmaxol and dieckol isolated from brown seaweed *E. maxima* could be considered effective anti-inflammatory agents as pharmaceutical and functional food ingredients.

## 1. Introduction

Attempts have been made to obtain bioactive materials from marine sources that are useful for human consumption [1,2]. Recently, the development of various materials as edible, medicinal, and cosmetics from various marine resources has attracted attention, and various marine-based products have gained great popularity among consumers [3]. Among marine organisms, seaweeds contain numerous biologically active natural products, including terpenoids, sulfated polysaccharides, peptides, polyunsaturated fatty acids, polyphenols, alkaloids, and mycosporine-like amino acids [4]. The brown algae *Ecklonia cava* has been extensively studied and found to contain phenolic compounds such as phlorotannin. Moreover, numerous bioactive studies on its antioxidant, anticancer, hepatoprotective, anti-inflammatory, antidiabetic, and anti-obesity properties have been conducted [5,6,7,8,9,10]. In this study, *Ecklonia maxima*, a species similar to *E. cava*, was examined.

*E. maxima* is a seaweed that is widely distributed on the west coast of South Africa. It is a brown alga, and research on this species is insufficient [11]. *E. maxima* grows up to 15 m or more, and 6000 tons per year has been supplied to the abalone farming industry [12]. Recently, the distribution of *E. maxima* on the coast of South Africa has been increasing, but it is difficult to utilize this resource as only a few studies have been conducted on it [11]. In contrast, numerous studies have been conducted on a related *Ecklonia* species, *E. cava*, and many products have been released as functional foods and cosmetics. Therefore, the study of *E. maxima*, which is expected to contain secondary metabolites similar to those of *E. cava*, should be carried out to investigate it for academic and industrial applications.

Inflammatory reactions are the cause of many diseases, and reducing inflammatory responses is very important to human health [13,14]. Recently, research using zebrafish, which have many genes that are conserved with mammals and are useful for in vivo studies, have been utilized for studies related to human health [15,16]. Research using zebrafish has been applied in various fields, including the reduction of the inflammatory response [17,18]. The anti-inflammatory response can be investigated by inducing an inflammatory reaction in zebrafish using lipopolysaccharide (LPS), tumor necrosis factor (TNF)-α, or interferon (IFN)-γ [17]. Induction of inflammation in zebrafish induces the generation of reactive oxygen species (ROS), nitric oxide (NO), and, subsequently, cell death. We evaluated the anti-inflammatory activity of extracts from *E. maxima*, specifically the reduction of ROS, NO production, and cell death in zebrafish induced by LPS. NO is an indicator of typical anti-inflammatory activity, and inhibition of NO production has been evaluated in many anti-inflammatory studies [2].

High-performance centrifugal partition chromatography (HPCPC) is a liquid–liquid chromatography method that can compensate for the disadvantages of conventional column chromatography technology for extracting natural products from various organisms [19]. The disadvantages of the existing methods are that they take a lot of time to extract natural products, they can involve severe sample loss, and there are issues with their reproducibility. However, HPCPC use solves these problems, as it has a short separation time, minimal sample loss, excellent reproducibility, and uses very little solvent [20].

Therefore, in this study, we aimed to isolate natural products from *E. maxima* with an efficient and simple process involving HPCPC. In addition, the structure of natural products was solved through liquid chromatography–mass spectrometry (LC/MS) and other analytical methods, and their anti-inflammatory activity was explored in a zebrafish embryo model.

## 2. Results

### 2.1. Isolation and Characterization of Phlorotannins from EEM

**Figure 1:**
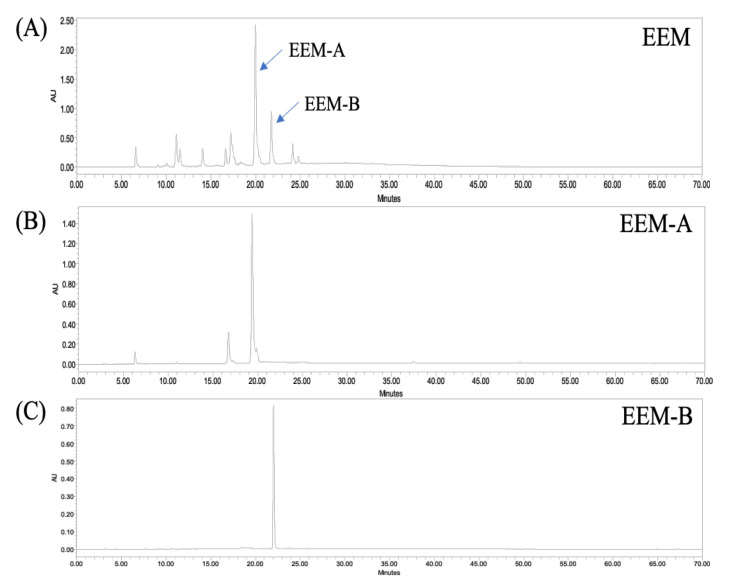
HPLC chromatogram of (**A**) EEM, (**B**) EEM-A, and (**C**) EEM-B.

In previous studies, both dieckol and eckmaxol were isolated from the EA fraction [21,22]. Both dieckol and eckmaxol have been reported to be useful because of their bioactivities, such as protective effects against oxidative stress, inflammatory mediator inhibitory effects, antidiabetic, and antibiotic effects. [1,5,6,7,9,10,21,23]. Various studies have been conducted to obtain these two compounds efficiently. However, isolating dieckol is difficult and requires successive steps, high solvent consumption, a long amount of time, and passive problems [24]. Therefore, we identified two compounds in EEM through HPLC analysis and set the HPCPC conditions to separate the two compounds for one-step purification (Figure 1A). As a result of analyzing the EEM, the main peak (EEM-A) was confirmed at a retention time (RT) of 20 min, and the second main peak (EEM-B) was confirmed at an RT of 22 min (Figure 1A). Solvent systems (n-hexane:EtOAc:MeOH:water, *v*/*v*) were tested at various ratios to investigate the optimal partition coefficient for two secure high-purity target compounds from EEM. As shown in Table 1, 2:7:3:7 of n-hexane:EtOAc:MeOH:water (*v*/*v*) among the several solvent systems tested was identified as the optimal solvent coefficient for the two target compounds. HPCPC was operated in the descending mode because the solvent partition coefficient of the identified solvent system was close to 1. A partition coefficient close to 1 has been shown to produce excellent compound resolution in previous studies [25,26,27,28]. The upper layer was used as the stationary phase, and the lower layer was used as the mobile phase. Because the partition coefficients of the two compounds are 0.94 and 1.41, the two compounds were able to be separated without overlapping (Table 1).

**Figure 2:**
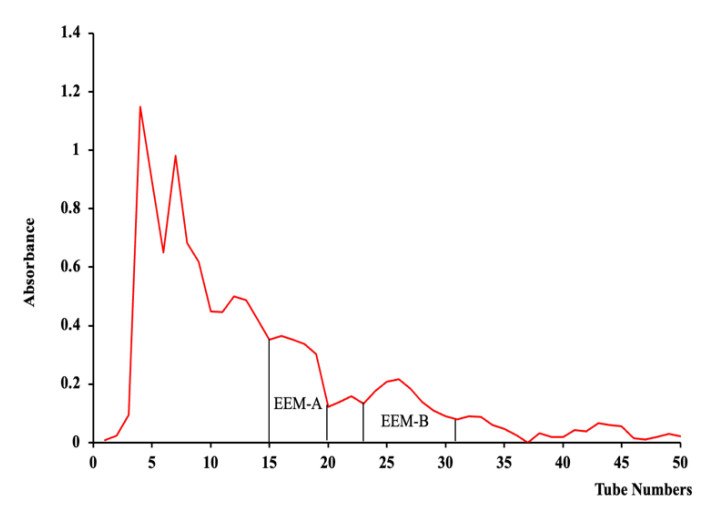
HPCPC chromatogram of EEM.

As a result of running comprehensive peak characterization using the conditions specified above, we confirmed that the target compounds, EEM-A and EEM-B, were separated without overlap because they had a 15 min difference in retention time (Figure 2).

**Figure 3:**
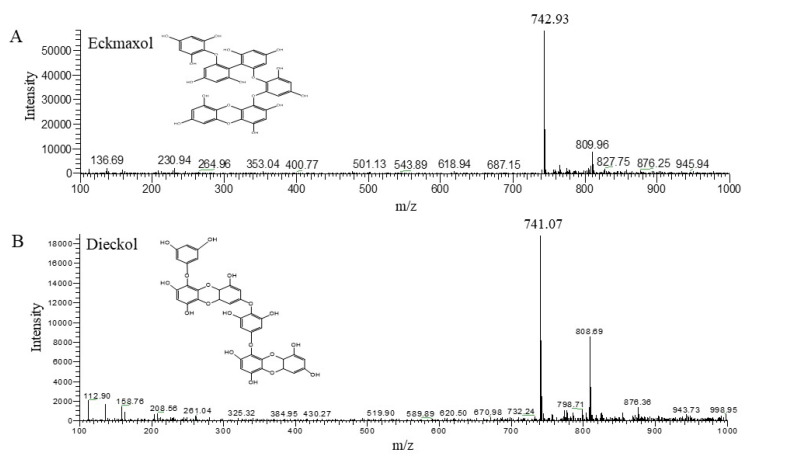
MS spectrum of (**A**) eckmaxol and (**B**) dieckol.

The isolated compounds were characterized by HPLC and ESI/MS. HPLC analysis confirmed that EEM-A and EEM-B were separated with high purity through comprehensive peak characterization (Figure 1B,C). The purity of the eckmaxol was recorded as more than 90%, and the same for the dieckol was recorded as more than 98%. After concentrating each fraction, 10 mg from each compound could be obtained. ESI/MS analysis based on the HPLC results revealed that EEM-A has a molecular weight of 743 and was confirmed to be eckmaxol, and EEM-B has a molecular weight of 742 and was confirmed to be dieckol (Figure 3). Cross comparison of the MS/MS data with previous publications indicated that the data are perfectly aligned with the obtained results. NMR analysis data are represented under the supplementary materials (Figure S3, Supplementary Material).

### 2.2. Protective Effect of EEM on Heartbeat Rates and Survival Rates in LPS-Induced Zebrafish Embryos

**Figure 4:**
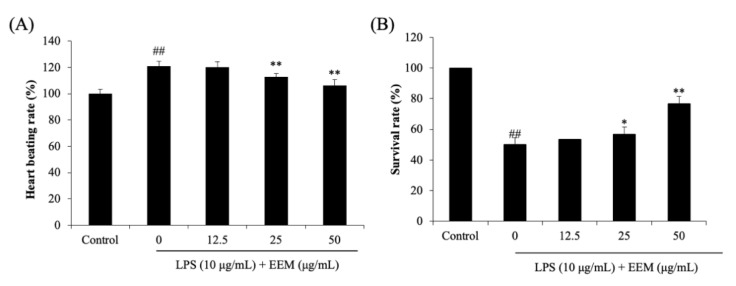
(**A**) The heart beating rate and (**B**) survival rate of LPS-stimulated zebrafish larvae. All values presented as the mean ± standard deviation (SD), *n* = 15. ## *p* < 0.01 vs. control group; * *p* < 0.05, and ** *p* < 0.01 vs. LPS-treated group.

Zebrafish have an organ system and physiology that is similar to humans, and so they are widely used as an in vivo animal model in many studies [29,30,31]. Heartbeat and survival rates were analyzed in LPS-stimulated zebrafish larvae, and EEM was found to have a protective effect. Zebrafish larvae treated with LPS showed a significantly increased heartbeat rate compared with the control group (Figure 4A). In the EEM-treated group, a significant decrease was observed at 25 and 50 μg/mL, but no significant difference was observed at the lowest concentration of 12.5 μg/mL (Figure 4A). The LPS-treated group showed significantly lower survival rates than the control group. However, the group treated with 50 μg/mL EEM showed a significant increase in survival rate. These results show that LPS induces an inflammatory response in zebrafish that results in an increased heartbeat rate and decreased survival. The survival rate was rescued by treatment with EEM, which likely interfered with the inflammatory response. Hence, we conducted an experiment to identify inflammation-related factors at EEM concentrations of 12.5, 25, and 50 μg/mL.

### 2.3. Inhibitory Effect of EEM on LPS-Induced ROS Generation, NO Production, and Cell Death

**Figure 5:**
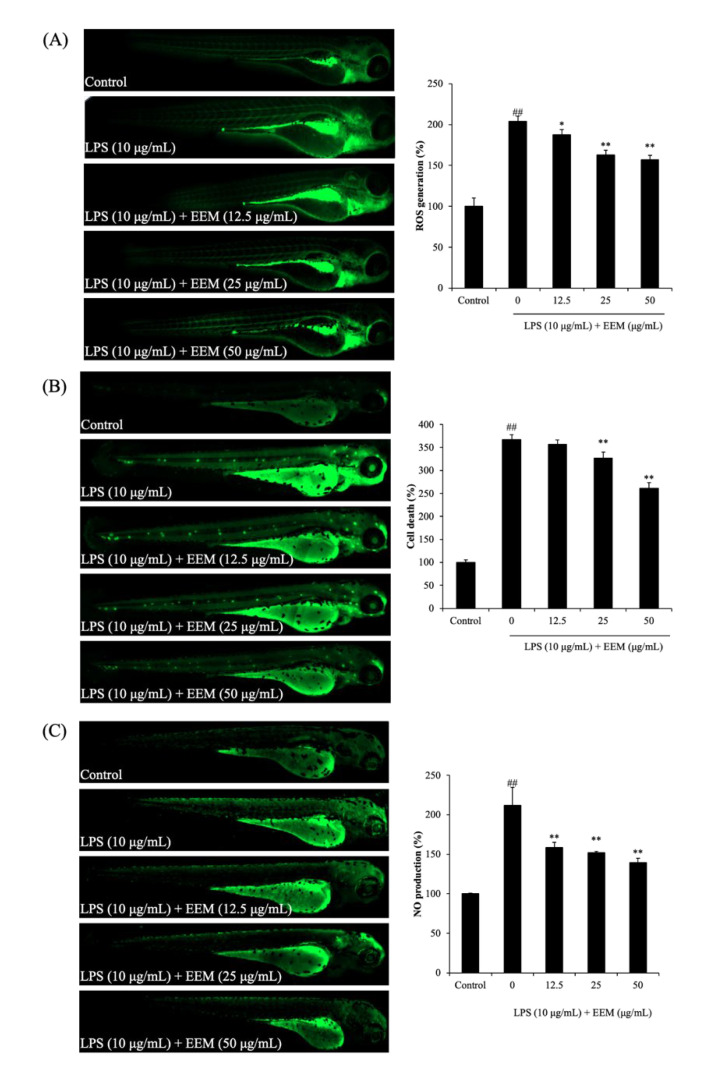
Effect of EEM on inflammation response in LPS-induced zebrafish. (**A**) ROS levels, (**B**) Cell death, and (**C**) NO production of LPS-stimulated zebrafish. Zebrafish embryos of 7–9 hpf were treated with EEM (12.5, 25, 50 µg/mL) and stimulated with LPS (10 µg/mL) up to 24 hpf. After that, ROS, cell death, and NO levels were measured through zebrafish larvae by DCFH2-DA, acridine orange, and DAF-FM-DA staining. The relative amounts of ROS, cell death, and NO of zebrafish were analyzed using Image J software. All values presented as the mean ± standard deviation (SD), *n* = 4. ## *p* < 0.01 vs. control group; * *p* < 0.05, and ** *p* < 0.01 vs. LPS-treated group.

ROS generation, cell death, and NO production in zebrafish larvae treated with LPS and EEM are shown in Figure 5. As shown in Figure 5A, the ROS generation in zebrafish larvae induced by LPS increased to 203.88% compared with the control group. The EEM-treated group significantly reduced the ROS generation induced by LPS, with the lowest ROS generation (156.92%) observed at the highest concentration of 50 µg/mL. Cell death was observed to be caused by the LPS inflammatory reaction in zebrafish larvae (Figure 5B). Treatment with EEM resulted in a significant decrease at 25 and 50 µg/mL, but there was no significant difference at 12.5 µg/mL. NO is an inflammatory factor, and we found that NO production was significantly increased in LPS-treated zebrafish larvae. EEM treatment caused a dose-dependent decrease, with the NO level reduced to 158.58%, 152.02%, and 139.53% for the 12.5, 25, and 50 µg/mL concentrations, respectively. These results demonstrate that EEM has anti-inflammatory effects by reducing ROS generation, cell death, and NO production in the inflammatory response caused by LPS in zebrafish.

## 3. Discussion

Numerous studies have been performed on the isolation of dieckol and eckmaxol, but none have been performed on the isolation of these two compounds from *E. maxima*. This study mainly establishes the technology and protocol for refining these two compounds, which have been reported to have excellent functionality in various studies, from *E. maxima* in a single process. In addition, by evaluating the anti-inflammatory activity of the EA fraction in zebrafish, the two compounds were identified as the main peaks confirming the functionality of the molecules isolated from the brown algae *E. maxima*. Obtaining effective compounds in one step through a simple process represents a great advantage for industrial and academic uses.

Seaweeds are known to exhibit various biological activities [22]. *Ecklonia* species have been investigated in several studies by many researchers [1,5,6,22,23]. As there are many reports of related secondary metabolites and their biological activities, they are considered to be suitable marine organisms for industrialization. *E. maxima*-related efficacy reported to date includes antioxidant, antineuroprotective, antimelanogenic, and antidiabetic effects [32,33,34,35]. However, compared with *E. cava*, which is a similar species, research on *E. maxima* is insufficient. In the case of *E. cava*, various effects have been confirmed in many studies, and it is used as a raw material for various industrial products. As the popularity of *E. cava* as a raw material for various products has increased, the supply of *E. cava* has recently not been able to meet the demand, so it is important to find alternative resources. *E. maxima* is a suitable resource to replace *E. cava* because it is widely distributed in South Africa, and it contains eckmaxol and dieckol, the compounds investigated in this study, which exhibit various biological activities.

Inflammation is a complex and important defensive host response, and in most cases, it is an autoimmune response to resolve infections or heal wounds [36]. However, in uncontrolled inflammatory reactions, ROS and several proteases may be produced, leading to chronic inflammation [37]. Previous studies have shown that polyphenols derived from algae have anti-inflammatory activity [38,39,40]. In this study, the potential anti-inflammatory properties of EEM isolated with 80% MeOH extraction were investigated. Chemicals derived from EEM inhibited LPS-induced inflammatory responses in animal model zebrafish larvae. The zebrafish larvae that were treated with EEM containing dieckol and eckmaxol showed a return to the control zebrafish larvae heartbeat rate and a recovery in the survival rate. These results are similar to studies on anti-inflammatory activity in zebrafish treated with seaweed extracts [8,41]. In addition, EEM significantly lowered ROS generation and cell death in zebrafish larvae induced by the inflammatory response to LPS, and dose-dependent NO production was observed. Previous studies used a higher concentration (100 μg/mL) than we used in our study, which indicates that the two high-purity polyphenol compounds (dieckol and eckmaxol) are present and show excellent activity [8]. Therefore, in this study, we isolated eckmaxol and dieckol, which are reported to have various physiological activities and could act synergistically, and suggest that they can be investigated as agents that are available to the pharmaceutical and functional food sectors in the future.

## 4. Materials and Methods

### 4.1. Materials

*E. maxima* samples were collected along the coast of Cape Town, Republic of South Africa in January 2019. Salt, sand, and epiphytes were removed using tap water, and samples were stored in a freezer. LPS, dimethyl sulfoxide (DMSO), phosphate-buffered saline (PBS), 2′-7′-dichlorodihydrofluorescein diacetate (DCF-DA), 4-amino-5-methylamino-2′-7′-difluorofluorescein diacetate (DAF-FM-DA), and acridine orange were obtained from Sigma-Aldrich (St. Louis, MO, USA). All other chemicals and reagents were analytical grade.

### 4.2. Preparation of Ethyl Acetate Fraction of E. maxima (EEM) and Isolation of Phlorotannins

*E. maxima* powder was extracted at room temperature (25–30 °C) using 80% MeOH, and the extract was evaporated with a solvent using a rotary evaporator. The crude extract was homogenized in an appropriate volume of water, then the same proportions of solvents, n-hexane, chloroform, and ethyl acetate (EA), were added, and samples were fractionated. Previously, many studies have reported dieckol separation studies derived from the EA fraction of *E. cava*, and studies have been conducted to investigate the various physiological activities of dieckol [9,10,23]. However, there have been no reports on dieckol purification from *E. maxima*. Furthermore, several reports have been published on the extraction of eckmaxol from *E. maxima* [21,32]. This study is the first report of eckmaxol and dieckol extraction and identification from *E. maxima*. Phlorotannins were purified by a simple process using HPCPC, which was conducted according to the method described by Kim et al. [19]. HPCPC was carried out using two solvent systems, which were divided by mixing n-hexane, ethyl acetate, methanol, and distilled water (DW). A previous study confirmed that the closer the solvent coefficient (*K*-value) was to 1, the better the separation effect achieved in the descending mode [19,42]. As a result of checking the solvent coefficients of the targeted eckmaxol and dieckol, using various solvent conditions to set the optimal solvent conditions, the ratio of 2:7:3:7 (*v*/*v*) of n-hexane:EA:MeOH:DW showed the best result (Table 1). The upper solvent phase was used as the stationary phase, and the lower solvent phase was used as the mobile phase for the separation of phlorotannins through the descending mode in an immiscible solvent system. The HPCPC column of the stationary phase was set to 1000 rpm, and the mobile phase was passed through the column at a flow rate of 2 mL/min. The EA fraction (300 mg) from *E. maxima* was dissolved in 6 mL of a 1:1 (*v*/*v*) mixture of the two immiscible solvents and injected into the injection valve. The separated fractions were collected using a fraction collector (FC 203 B, Gilson, South Korea), and the process was monitored under 290 nm ultraviolet (UV) light.

### 4.3. HPLC-PDA-ESI/MS Analysis of Phlorotannins

The analysis was performed using a high-pressure liquid chromatography-photodiode array (HPLC-PDA) Accela system (Thermo Scientific, San Jose, CA, USA) coupled with an LCQ Fleet ion trap mass spectrometer (ESI/MS, Thermo Scientific, San Jose, CA, USA). Both positive and negative ion mass spectra were recorded in the range m/z 100–1000. The source voltage was set to 5 kV, and the capillary temperature was 275 °C. The capillary voltage was maintained at 42 V, the tube lens at 125 V, and the sheath gas at 30 μL/min.

### 4.4. In Vivo Zebrafish Embryo Model

#### 4.4.1. Application of EEM and LPS to Zebrafish Embryos

Zebrafish embryo experiments were performed using an assay described by Kim et al. [19]. In brief, embryos at approximately 7–9 h postfertilization (hpf) (*n* = 15) were transferred to individual wells of a 12-well plate containing 900 μL embryonic medium. Next, 50 μL of each well was treated with 10 μg/mL LPS and various concentrations of EEM. Embryos were incubated in 10 μg/mL LPS solution for 1 h before being exposed to varying concentrations of EEM. After the addition of EEM, embryos were incubated for 24 h and then rinsed with fresh media. The animal study protocol was approved by the Institutional Animal Care and Use Committee (IACUC) of the Jeju National University Animal Center (Approval Number. 2020-0049).

#### 4.4.2. Measurement of Heartbeat and Survival Rate

Heartbeat and survival rates were measured following the protocol described by Kim et al. [19]. The heartbeat was measured at 48 hpf. Atrial and ventricular conditions were monitored under a microscope for 1 min, and the results are expressed as the mean heartbeat rate per minute. The survival rate was measured at 72 hpf.

#### 4.4.3. Measurement of ROS Generation, NO Production, and Cell Death via Image Analysis in Zebrafish Embryos

Intracellular ROS generation, NO production, and cell death in zebrafish embryos were determined according to methods described by Lee et al. [43]. To assess cell death, zebrafish embryos were transferred into 12-well plates and treated with acridine orange solution (7 μg/mL), and the plates were incubated for 30 min in the dark at 28.5 °C. To quantify ROS and NO generation, zebrafish embryos were treated with DCF-DA solution (20 μg/mL) and DAF-FM-DA solution (5 μM), respectively, and incubated for 1 h.

### 4.5. Statistical Analysis

All experiments were performed in triplicate, and all values are presented as mean ± standard error. Analysis of variance (ANOVA) and Tukey post-hoc tests were performed with the IBM SPSS Statistics 20 software (Chicago, IL, USA). Results were considered statistically significant at *p* < 0.05.

## 5. Conclusions

In this study, the anti-inflammatory effects of EEM derived from *E. maxima* containing dieckol and eckmaxol in zebrafish larvae were investigated. EEM, containing a large amount of polyphenol, was separated using 80% MeOH extraction from *E. maxima*. We isolated high-purity EEM-A and EEM-B in a relatively simple manner using HPCPC, and dieckol and eckmaxol were analyzed. Subsequently, the LPS-induced zebrafish larvae were treated with EEM containing dieckol and eckmaxol to examine their anti-inflammatory effects. EEM showed protective effects on heartbeat and survival rates in zebrafish larvae. In addition, the anti-inflammatory effect was verified by the reduction of ROS generation, cell death, and NO production. Therefore, dieckol and eckmaxol isolated from *E. maxima* through HPCPC can be considered effective anti-inflammatory agents for pharmaceutical and functional food ingredients.

## Figures and Tables

**Table 1:** The K (partition coefficient) values of eckmaxol and dieckol in the two-phase solvent systems.

Solvent Condition		*K-*Value	
		Eckmaxol	Dieckol
Hexane:EtOAc:MeOH:Water	1:9:3:7	2.59	5.94
	2:7:3:7	0.94	1.41
	2:8:3:7	1.16	2.34
	2:8:4:6	0.35	0.37
	3:7:3:7	0.23	0.29

## Data Availability

Not applicable.

## Supplementary Materials

The following supporting information can be downloaded at: https://www.mdpi.com/article/10.3390/md20080471/s1, Figure S1: LC/MSMS spectra (negative ion mode) of eckmaxol; Figure S2: LC/MSMS spectra (negative ion mode) of dieckol; Figure S3: NMR spectroscopic data of eckmaxol [32] and dieckol [44]; Figure S4: Effect of eckmaxol on inflammation response in LPS-induced RAW 264.7 cells. (A) Cell viability and NO production and (B) IL-6 and IL-1β of LPS-stimulated RAW 264.7 cells.
