# Supplementary material for: Isolation and Characterization of Efficient Active Compounds Using High-Performance Centrifugal Partition Chromatography (CPC) from Anti-Inflammatory Activity Fraction of Ecklonia maxima in South Africa
Source: Mar Drugs. 2022 Jul 23;20(8):471. doi: 10.3390/md20080471 (PMC9394317; doi:10.3390/md20080471)
Supplement: Supplementary file 1 [file marinedrugs-20-00471-s001.zip › marinedrugs-1826115-supplementary.pdf]

Supplementary data

Isolation and characterization of efficient active compounds using high-performance centrifugal partition chromatography (CPC) from anti-inflammatory activity fraction of *Ecklonia maxima* in South Africa

Supplementary data

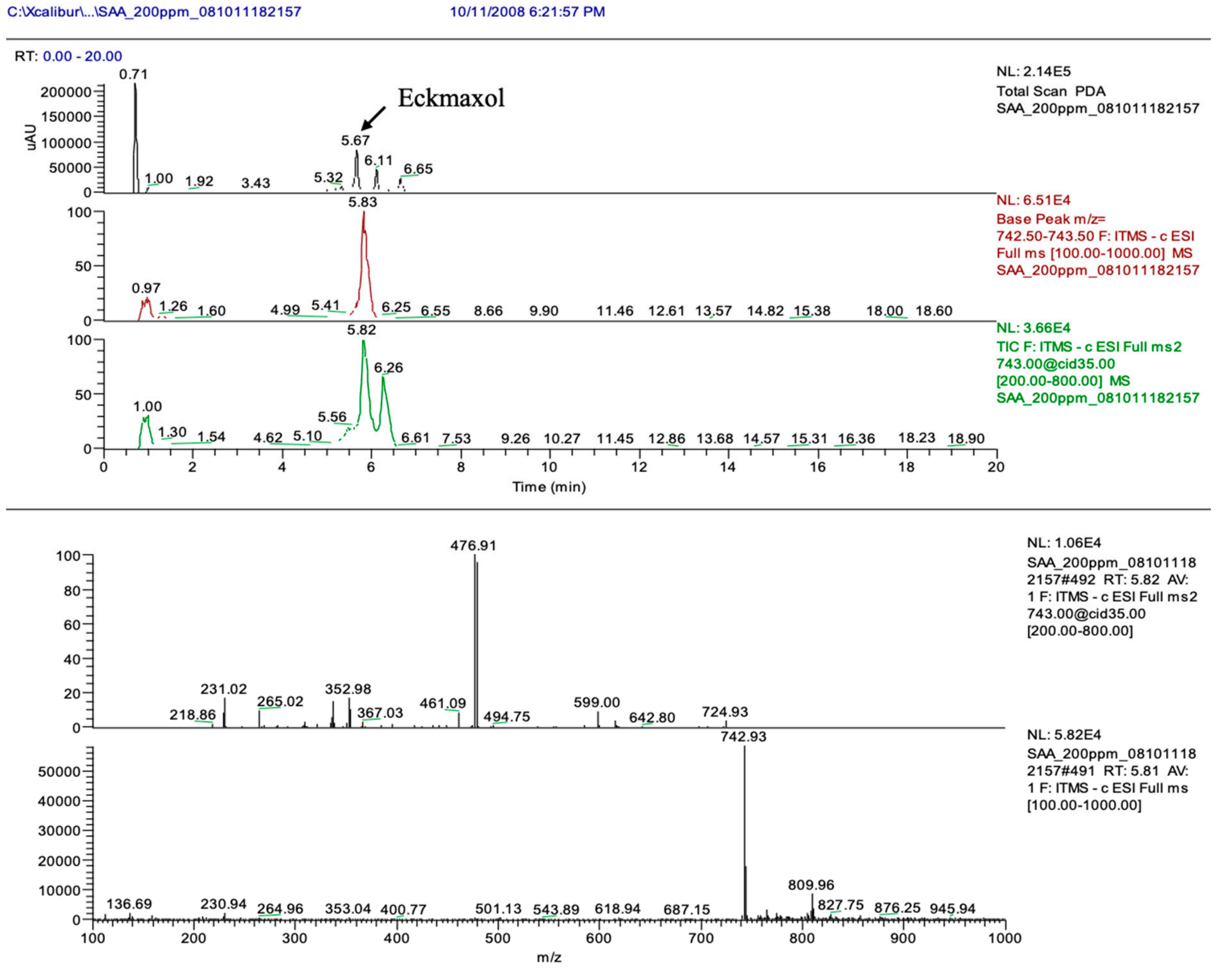

Supplemental Figure 1. LC/MSMS spectra (negative ion mode) of Eckmaxol

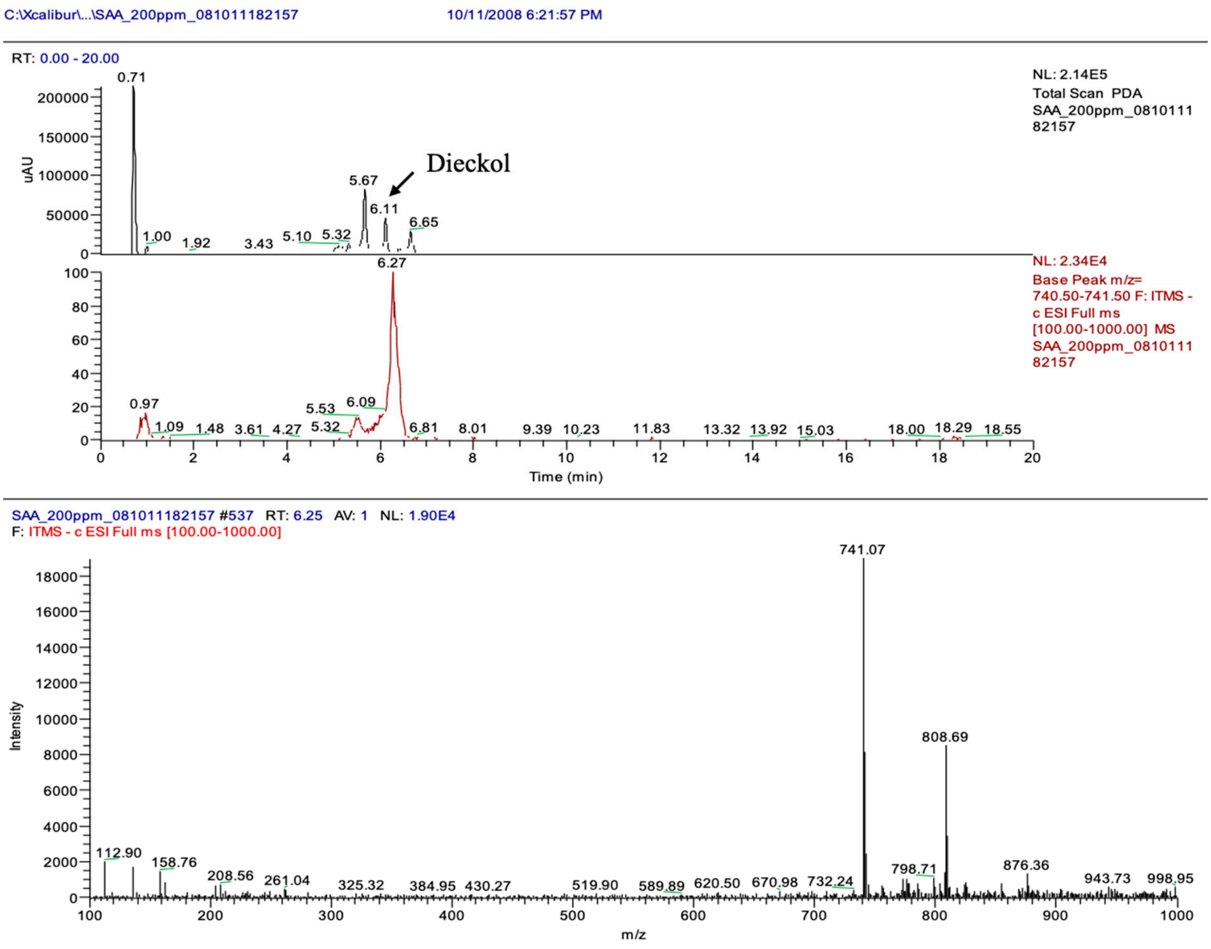

Supplemental Figure 2. LC/MSMS spectra (negative ion mode) of Dieckol

(A) Eckmaxol

Pos.	δH	δC	Pos.	δH	δC
1		124.8	19		157.1
2		151.3	20	5.93 (1H, d, *J* = 2.4 Hz, H-20)	94.2
3	6.11 (1H, d, *J* = 2.4 Hz, H-3)	97.6	21		159.2
4		156.4	22	6.32 (1H, d, *J* = 2.4 Hz, H-22)	98.7
5	5.86 (1H, d, *J* = 2.4 Hz, H-3)	94.7	23		143.3
6		154.4	24		102.0
7		123.5	25		101.8
8		137.7	26		156.1
9		143.5	27	6.20 (1H, d, *J* = 2.0 Hz, H-27)	95.2
10	6.20 (1H, s, H-10)	98.9	28		159.3
11		146.3	29	6.28 (1H, d, *J* = 2.0 Hz, H-29)	98.3
12		124.7	30		158.5
13		153.4	31		124.0
14	6.01 (1H, d, *J* = 2.4 Hz, H-14)	99.3	32		151.6
15		158.7	33	6.03 (1H, s, H-33)	96.1
16	5.94 (1H, d, *J* = 2.4 Hz, H-16)	95.2	34		156.1
17		146.7	35	6.03 (1H, s, H-35)	96.1
18		123.8	36		151.6

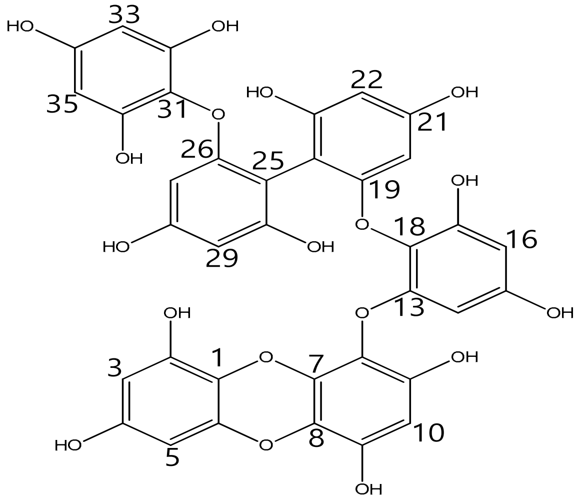

Chemical Formula: $C_{36}H_{24}O_{18}$
Molecular Weight: 744.56

(B)

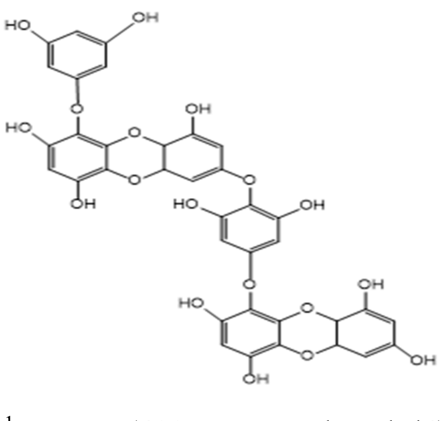

Dieckol: amorphous powder, ^{1}H NMR (400 MHz, methanol-d4) δ 6.15 (1H,s), 6.13 (1H, s), 6.09 (1H, d, 2.9 Hz), 6.06 (1H, d, 2.9 Hz), 6.05 (1H, d, 2.9 Hz), 5.98 (1H, d, 2.8 Hz), 5.95 (1H, d, 2.8 Hz), 5.92 (3H, m); ^{13}C NMR (100 MHz, Methanol-d4) δ 161.8, 160.1, 157.8, 155.9, 154.5, 152.4, 147.3, 147.2, 147.1, 146.9, 144.3, 144.1, 143.4, 143.3, 138.6, 126.5,

126.2, 125.6, 125.5, 124.9, 124.6, 124.5, 99.9, 99.7, 99.5, 99.4, 97.6, 96.2, 95.8, 95.7, 95.3; FAB-MS m/z 741 [M]-.

Supplemental Figure 3. NMR spectroscopic data of Eckmaxol [32] and Dieckol [44].

(A)

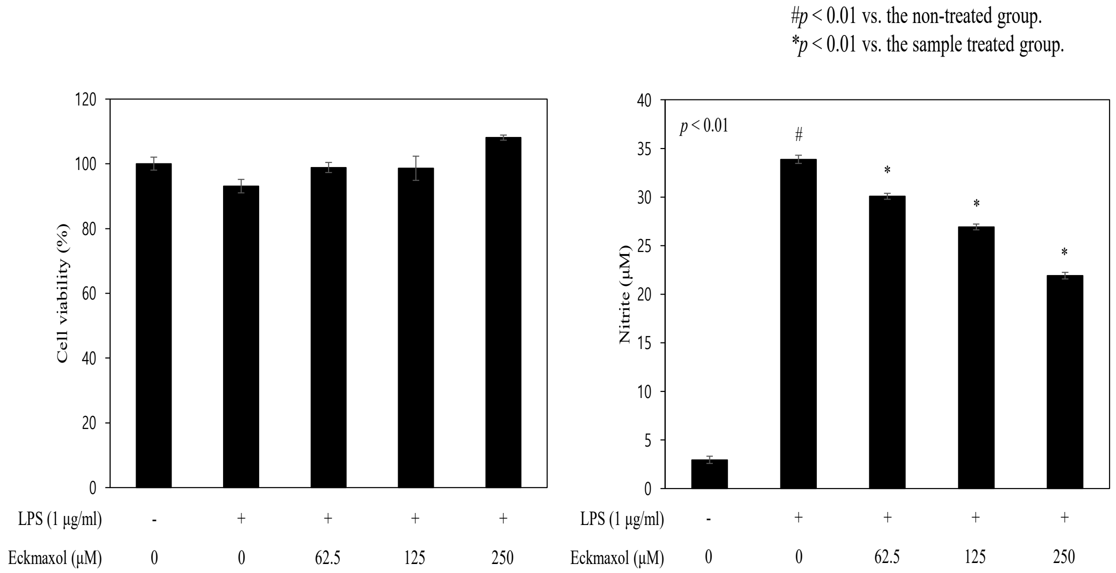

(B)

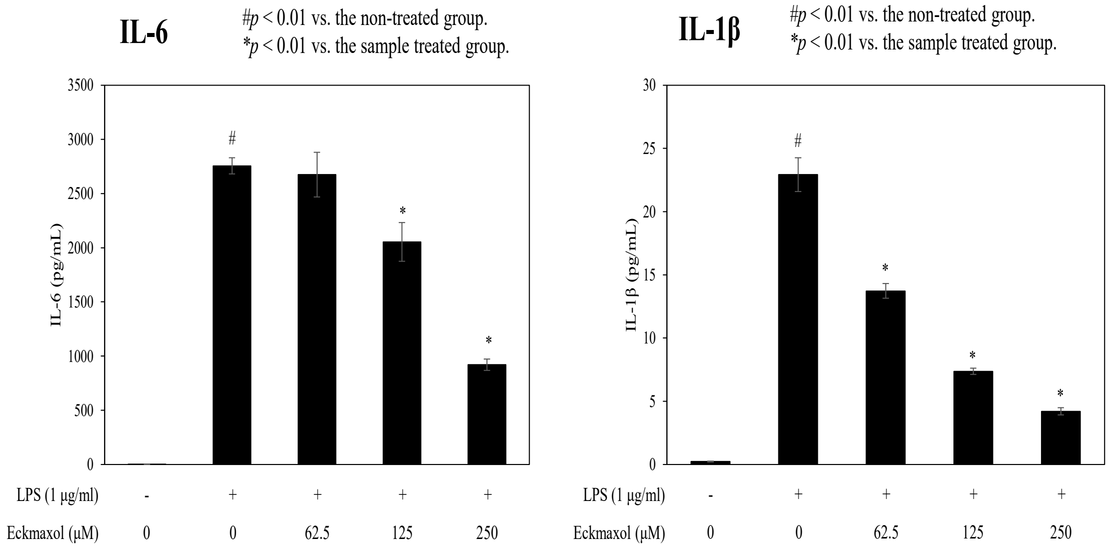

Supplemental Figure 4. Effect of Eckmaxol on inflammation response in LPS-induced RAW 264.7 cells. (A) Cell viability and NO production and (B) IL-6 and IL-1β of LPS-stimulated RAW 264.7 cells.

Reference

32 Zhou X, Yi M, Ding L, He S, Yan X. Isolation and purification of neuroprotective phlorotannin from the marine Algae Ecklonia maxima by size exclusion and high-speed counter-current chromaxography. Marine drugs. 2019, 17, 212.

44 Lee SH, Han JS, Heo SJ, Hwang JY, Jeon YJ. Protective effects of dieckol isolated from Ecklonia cava against high glucose-induced oxidative stress in human umbilical vein endothelial cells. Toxicology in Vitro. 2010, 24, 375–381.
